# Bok: real killer or bystander with non-apoptotic roles?

**DOI:** 10.3389/fcell.2023.1161910

**Published:** 2023-04-12

**Authors:** Caden G. Bonzerato, Richard J. H. Wojcikiewicz

**Affiliations:** Department of Pharmacology, SUNY Upstate Medical University, Syracuse, NY, United States

**Keywords:** Bcl-2 related ovarian killer, ubiquitin-proteasome pathway, mitochondrial dynamics, mitochondria-associated ER membranes, apoptosis, myeloid-cell leukemia 1, inositol 1 4 5-trisphosphate receptor

## Abstract

Bcl-2-related ovarian killer, Bok, was first labeled “pro-apoptotic” due to its ability to cause cell death when over-expressed. However, it has become apparent that this is not a good name, since Bok is widely expressed in tissues other than ovaries. Further, there is serious doubt as to whether Bok is a real “killer,” due to disparities in the ability of over-expressed versus endogenous Bok to trigger apoptosis. In this brief review, we rationalize these disparities and argue that endogenous Bok is very different from the pro-apoptotic, mitochondrial outer membrane permeabilization mediators, Bak and Bax. Instead, Bok is a stable, endoplasmic reticulum-located protein bound to inositol 1,4,5 trisphosphate receptors. From this location, Bok plays a variety of roles, including regulation of endoplasmic reticulum/mitochondria contact sites and mitochondrial dynamics. Therefore, categorizing Bok as a “killer” may well be misleading and instead, endogenous Bok would better be considered an endoplasmic reticulum-located “bystander”, with non-apoptotic roles.

## Introduction

The Bcl-2 (B Cell lymphoma 2) protein family members are key regulators of the intrinsic (mitochondrial) apoptosis pathway (Vandenabeele et al., 2022). Since the discovery of Bcl-2 (Vaux et al., 1988), more than 15 family members have been characterized and divided into three subgroups based on their ability to initiate, promote, or suppress apoptosis, as well as the presence or absence of four conserved Bcl-2 homology (BH) domains, BH1-4 (Kale et al., 2018). The initiators of apoptosis are the BH3-only proteins, Bim, Bid, Puma, etc., and are activated in response to apoptotic stimuli. The promoters are the pro-apoptotic proteins, Bak and Bax, and contain BH1-4 domains. The suppressors are the anti-apoptotic proteins, Bcl-2, Bcl-xL, Mcl-1, etc., which also contain BH1-4 domains and antagonize pro-apoptotic family members (Kale et al., 2018; Moldoveanu and Czabotar, 2020). The balance between the activities of these proteins governs cell fate and homeostasis, with disruption of this Bcl-2 family network contributing to many diseases, from autoimmune and degenerative disorders to cancer (Xiang et al., 2018; Knight et al., 2019).

During intrinsic apoptosis signaling, Bak and Bax oligomerize to form pores that permeabilize the mitochondrial outer membrane (MOM), releasing proteins like cytochrome *c* into the cytosol, which in turn activate a downstream signaling cascade and ultimately cause cell death (Westphal et al., 2014). This irreversible process is known as mitochondrial outer membrane permeabilization (MOMP) and various studies have indicated that Bcl-2-related ovarian killer, Bok, might also be a MOMP mediator (Llambi et al., 2016; Fernandez-Marrero et al., 2017; Zheng et al., 2018; Shalaby et al., 2022). Indeed, in many reviews (Moldoveanu and Czabotar, 2020; Shalaby et al., 2020; Vandenabeele et al., 2022; Moldoveanu, 2023), Bok is grouped together with Bak and Bax as a *bona fide* MOMP mediator. However, this is hard to reconcile with the fact that endogenous Bok is predominately localized to the endoplasmic reticulum (ER) membrane, bound to inositol 1,4,5 trisphosphate receptors (IP_3_Rs) (Echeverry et al., 2013; Schulman et al., 2013; Schulman et al., 2016; Bonzerato et al., 2022). Here, we discuss the properties of Bok, in order to clarify whether it is truly a Bak/Bax-like “killer”, or an ER-located “bystander” with less-lethal roles.

## Bok as a “killer”

Bok was originally shown to be expressed in ovaries and strongly interact with some Bcl-2 family members (e.g., Mcl-1), but not others (e.g., Bak and Bax). It was categorized as a “killer” because over-expressed, “exogenous” Bok caused apoptosis (Hsu et al., 1997). Subsequent studies revealed that Bok is actually expressed in all cells and tissues (Ke et al., 2012; Naim and Kaufmann, 2020; Bonzerato et al., 2022) and confirmed that it can induce apoptosis when over-expressed in a variety of cell types (Echeverry et al., 2013; Einsele-Scholz et al., 2016; Llambi et al., 2016; Schulman et al., 2016; Szczesniak et al., 2021b). Also, cell-free experiments showed that Bok can permeabilize liposomes or artificial MOMs, like its pro-apoptotic counterparts Bak and Bax (Llambi et al., 2016; Fernandez-Marrero et al., 2017; Zheng et al., 2018; Shalaby et al., 2022). So, there is no doubt that over-expressed Bok in an exogenous context, or Bok in a cell-free system, can induce MOMP and apoptosis. But what about “endogenous” Bok in a native context? Bok knockout (KO) mice have no significant developmental or phenotypic abnormalities (Ke et al., 2012; Ke et al., 2013; Carpio et al., 2015; Llambi et al., 2016) and apoptotic responses are normal in various Bok KO cells, including mouse embryonic fibroblasts (MEFs) (Schulman et al., 2019), mouse lymphoid, myeloid, and cortical neurons (Ke et al., 2012; D'Orsi et al., 2016), Chinese hamster ovary cells (MacDonald et al., 2022), human leukemia (Flores-Romero et al., 2022) and synovial sarcoma cells (Muenchow et al., 2020). Since intrinsic apoptosis is dependent on the *bona fide* MOMP mediators Bak and Bax, Bok could potentially be acting redundantly with these proteins, yet current evidence suggests that Bok still plays little or no apoptotic role in Bak/Bax KO mice (Ke et al., 2022) and cells (Heimer et al., 2019; Bonzerato et al., 2022). However, some studies suggest that Bok KO protects against ER stress-induced apoptosis in MEFs (Carpio et al., 2015) and SH-SY5Y cells (Walter et al., 2022), although this is not always seen (Echeverry et al., 2013; Fernandez-Marrero et al., 2016; Schulman et al., 2019; Bonzerato et al., 2022). Further, several studies have indicated that acute depletion of Bok using siRNA can inhibit apoptotic signaling (Yakovlev et al., 2004; Jaaskelainen et al., 2010; Einsele-Scholz et al., 2016; Llambi et al., 2016), although this inhibitory effect of acute Bok depletion is not always observed (Fernandez-Marrero et al., 2016; Bonzerato et al., 2022) and possible off-target effects of the siRNAs are not given full consideration. It is possible though that acute Bok depletion, rather than prolonged Bok KO, is better able to reveal the apoptotic effects of Bok because the former approach provides less opportunity for adaptation. Overall, because of its constitutive binding to IP_3_Rs and the lack of effect of Bok KO, it is hard to conceive how endogenous Bok could be a canonical Bak/Bax-like MOMP mediator. Bok may play a role in ER stress signaling and apoptosis under certain conditions, but whether this is due to regulatory events at the ER, or at the MOM remains to be determined (Naim and Kaufmann, 2020).

So why the discrepancies between the MOMP-promoting properties of endogenous and exogenous Bok? It is most likely due to the fact that the vast majority of endogenous Bok is “locked-up” at the ER membrane by IP_3_R-binding and is simply not “free” to translocate to mitochondria to trigger MOMP (Naim and Kaufmann, 2020; Bonzerato et al., 2022). In contrast, over-expressed, exogenous Bok likely swamps the available IP_3_R binding sites at the ER and some Bok will be free to mediate MOMP directly, or perhaps perturb the balance between Bcl-2 family members; e.g., exogenous Bok could antagonize the activity of anti-apoptotic proteins, like Mcl-1, shifting the balance towards cell death. If endogenous Bok is never actually able to cause MOMP, it is perhaps surprising that purified Bok retains the ability to permeabilize liposomes or artificial MOMs in a cell-free system. This likely reflects the high degree of conservation between Bcl-2 family members, since Bak, Bax, and Bok have very similar structures and helix orientations required for pore formation (Ke et al., 2018; Zheng et al., 2018). Remarkably, even tBid, which is not usually thought of as a MOMP mediator, can directly permeabilize the MOM (Flores-Romero et al., 2022; Moldoveanu, 2023), indicating that the ability to cause MOMP is conserved between a variety of Bcl-2 family members.

Could there be conditions under which endogenous Bok is freed from IP_3_Rs and its killing potential unleashed? Available evidence indicates that this is not the case when endogenous Bok levels are normal, since neither apoptotic stimuli, nor IP_3_R-activation release Bok from IP_3_Rs (Schulman et al., 2016). However, upregulation of Bok expression could potentially swamp the available IP_3_R binding sites and lead to the creation of free Bok. This is plausible, as the Bok promoter can be activated by the E2F transcription factor family, leading to an upregulation of Bok mRNA (Rodriguez et al., 2006). Further, Bok mRNA stability can be regulated by microRNAs, leading to an increase in Bok protein levels and apoptosis (Onyeagucha et al., 2017; Hu et al., 2020; Naim and Kaufmann, 2020; Okazaki et al., 2020; Di et al., 2021). Whether such increases in Bok expression actually lead to the creation of free Bok remains to be determined.

## Bok as a “bystander”

So, if endogenous Bok is not a real killer, what is its role? An ER-localized bystander with non-apoptotic roles? Certainly, to understand these possible roles it will be necessary to consider the intimate relationship between Bok and IP_3_Rs and how Bok can influence events while confined at the ER membrane.

### Bok binding to IP_3_Rs and stability

IP_3_Rs (IP_3_R1, IP_3_R2, and IP_3_R3) are ER membrane proteins that form tetrameric channels and control the release of Ca^2+^ from the ER lumen into the cytosol (Prole and Taylor, 2019). Several Bcl-2 family members, e.g., Bcl-2 and Bcl-xL, interact with IP_3_Rs, but the interaction between Bok and IP_3_Rs is unique because 1) it is the only Bcl-2 family member that strongly co-immunoprecipitates with IP_3_Rs (Schulman et al., 2013), 2) the vast majority of endogenous Bok is constitutively bound to IP_3_Rs, with apparently one Bok protein bound to each IP_3_R subunit (Schulman et al., 2016; Schulman et al., 2019), and 3) the Bok/IP_3_R interaction is by far the strongest among Bcl-2 family members, with a K_d_ ∼65 nM, while Bcl-2 and Bcl-xL have K_d_ values of ∼1 µM and ∼0.7 µM, respectively (Monaco et al., 2012; Szczesniak et al., 2021a; Rosa et al., 2022). An unstructured and surface-exposed loop in IP_3_R1 has been shown to be the Bok binding site and interestingly, this loop is only conserved in IP_3_R1 and IP_3_R2, but not IP_3_R3, which correlates with the inability of IP_3_R3 to bind Bok (Schulman et al., 2013; Szczesniak et al., 2021a). Remarkably, Bok expression level is critically dependent upon IP_3_Rs, since deletion of IP_3_R1 and IP_3_R2 in MEFs reduced endogenous Bok levels by approximately 98%, and a similar dependence is seen in other (αT3, HeLa, DT40) cell lines (Schulman et al., 2019; Bonzerato et al., 2022). In the absence of IP_3_Rs, Bok is highly unstable and rapidly degraded by the ubiquitin-proteasome pathway (UPP) (Schulman et al., 2016; Bonzerato et al., 2022). These unique stability characteristics dictate that endogenous Bok is found at the ER membrane and suggest that whatever it does in the cell is achieved from this “bystander” location.

Surprisingly, endogenous and exogenous Bok are processed differently. A module including the E3 enzyme, gp78, ubiquitinates and degrades exogenous Bok, which accumulates and causes apoptosis during UPP-inhibition (Llambi et al., 2016). These data have been extrapolated to create a hypothesis that *endogenous* Bok is constitutively pro-apoptotic, but kept at very low, or “safe,” levels by the UPP (Llambi et al., 2016; Moldoveanu and Zheng, 2018). However, many other studies have shown that endogenous Bok is expressed at readily-detectable levels, gp78 is not involved in its processing, and UPP-inhibition does not increase endogenous Bok levels, or cause Bok-dependent apoptosis (Schulman et al., 2016; Moravcikova et al., 2017; Bonzerato et al., 2022). Thus, it seems that the hypothesis (Llambi et al., 2016) should be re-evaluated.

### Membrane insertion of Bok

**FIGURE 1:**
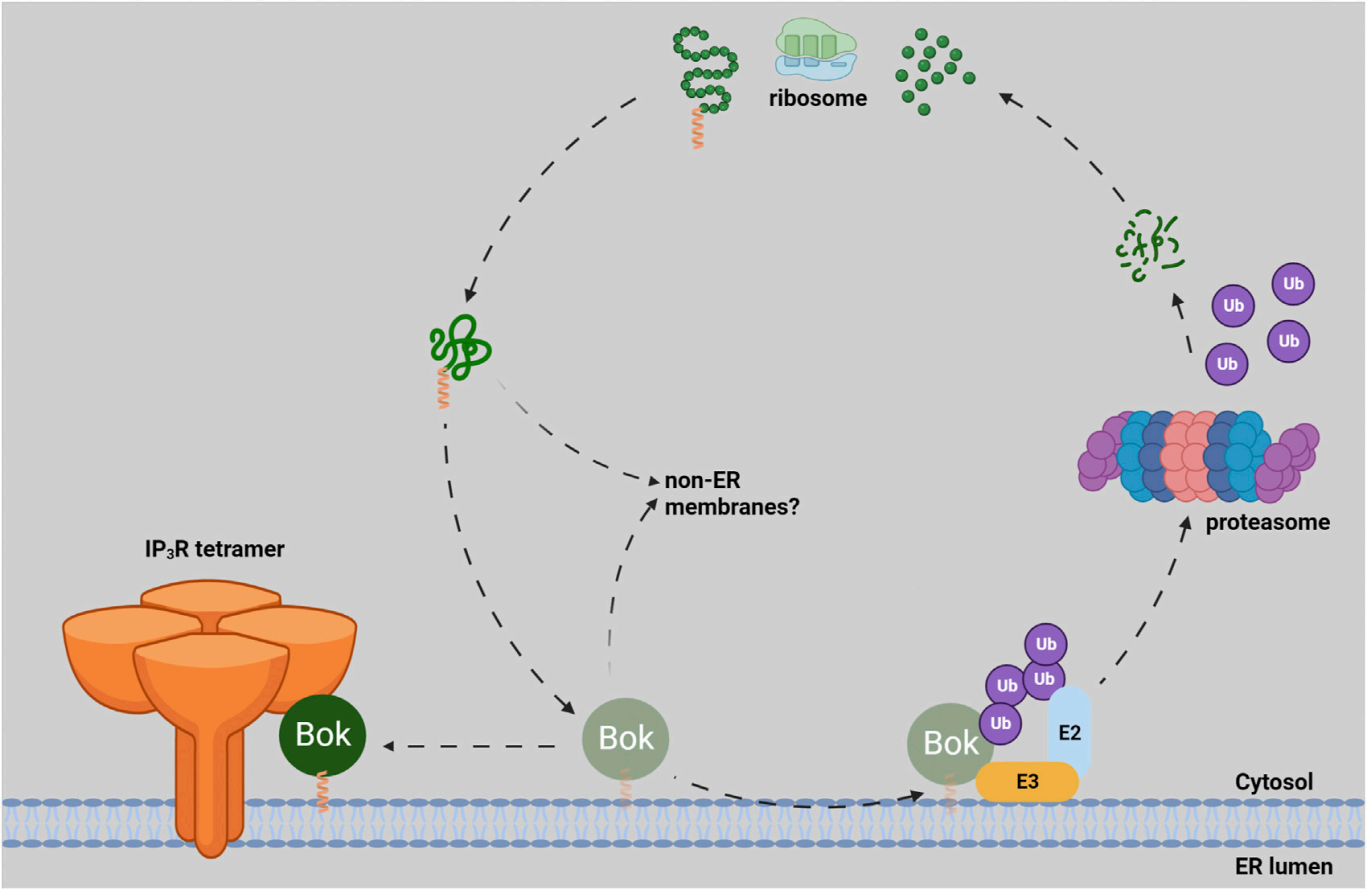
Lifecycle of endogenous Bok. Newly synthesized Bok, with its C-terminal TA (green polypeptide with orange helical TMD) emerges from the ribosome and is chaperoned by an unknown mechanism to the ER membrane. Once in the ER, Bok binds to IP_3_Rs and becomes stable. If Bok is unable to locate IP_3_Rs (e.g., in IP_3_R KO cells), Bok is unstable and rapidly degraded by the UPP. The ubiquitin-conjugating enzyme (E2) and the ubiquitin-ligase enzyme (E3) responsible for Bok ubiquitination (and subsequent delivery to the proteasome) are currently not defined (Bonzerato et al., 2022). It is possible that some Bok inserts into non-ER membranes, but in those cases, Bok would be unstable.

Contemplating the creation and destruction of Bok is an intriguing topic (Figure 1). Like almost all Bcl-2 family members, Bok is a tail-anchored (TA) protein, i.e., its transmembrane domain (TMD) is found at the very C-terminus (Lindsay et al., 2011). TA proteins are fully excluded from the ribosome and are transiently cytosolic before they are targeted to membranes, and several molecular mechanisms have been proposed to account for their delivery and insertion; e.g., the GET pathway for ER membrane targeting (Chio et al., 2017). Surprisingly, as none of these targeting mechanisms have been strongly linked to Bcl-2 family proteins, it remains unclear how they are delivered and inserted. The different subcellular localizations of Bcl-2 family members seem to be governed by certain features of their TMDs, like length and hydrophobicity, as well as flanking charged residues (Popgeorgiev et al., 2018; Jiang, 2021). However, the targeting rules are not strict, as individual Bcl-2 family members are found in multiple locations and they can move between membranes (Kale et al., 2018; Popgeorgiev et al., 2018). The most likely scenario for newly synthesized endogenous Bok is that it is initially guided to the ER membrane by the properties of its TMD and once it binds to IP_3_Rs, Bok accumulates there. If not bound to IP_3_Rs, endogenous Bok is subjected to “quality control” at the ER membrane and rapidly degraded by the UPP, although the E2 and E3 enzymes responsible have yet to be identified (Jiang, 2021; Bonzerato et al., 2022). Thus, essentially all endogenous Bok is found at the ER membrane (Figure 1). It is a possibility though, that a small proportion of newly synthesized Bok could insert into other membranes (e.g., into the MOM), but in those cases, Bok would not find IP_3_Rs, would be rapidly degraded, and would not accumulate.

### Does Bok regulate IP_3_R Ca^2+^ channel activity?

There is a vast body of literature indicating that Bcl-2 family members can regulate IP_3_R activity. For example, both Bcl-2 and Bcl-xL interact with IP_3_Rs and sensitize IP_3_R-mediated Ca^2+^ release from the ER (Monaco et al., 2012; Ivanova et al., 2020; Rosa et al., 2022). In view of the high affinity binding between Bok and IP_3_Rs, it is perhaps surprising that Bok does not substantially impact IP_3_R-mediated Ca^2+^ release. Cytosolic Ca^2+^ changes due to IP_3_R activation, measured using fluorescent Ca^2+^ indicator proteins or dyes, were not dependent on Bok (Schulman et al., 2013; Schulman et al., 2019; Szczesniak et al., 2021b; Carpio et al., 2021). Also, IP_3_R channel activity measured via electrophysiological methods was unaltered by Bok KO (Schulman et al., 2019). One study in primary cortical neurons demonstrated that early and prolonged cytosolic Ca^2+^ changes in response to N-methyl-D-aspartate were dependent on Bok (D’Orsi et al., 2016). However, this can be ascribed to regulation of glutamate receptor-mediated Ca^2+^ responses, rather than a direct effect on the activity of IP_3_Rs.

### Bok regulation of mitochondria-associated ER membranes (MAMs)

**FIGURE 2:**
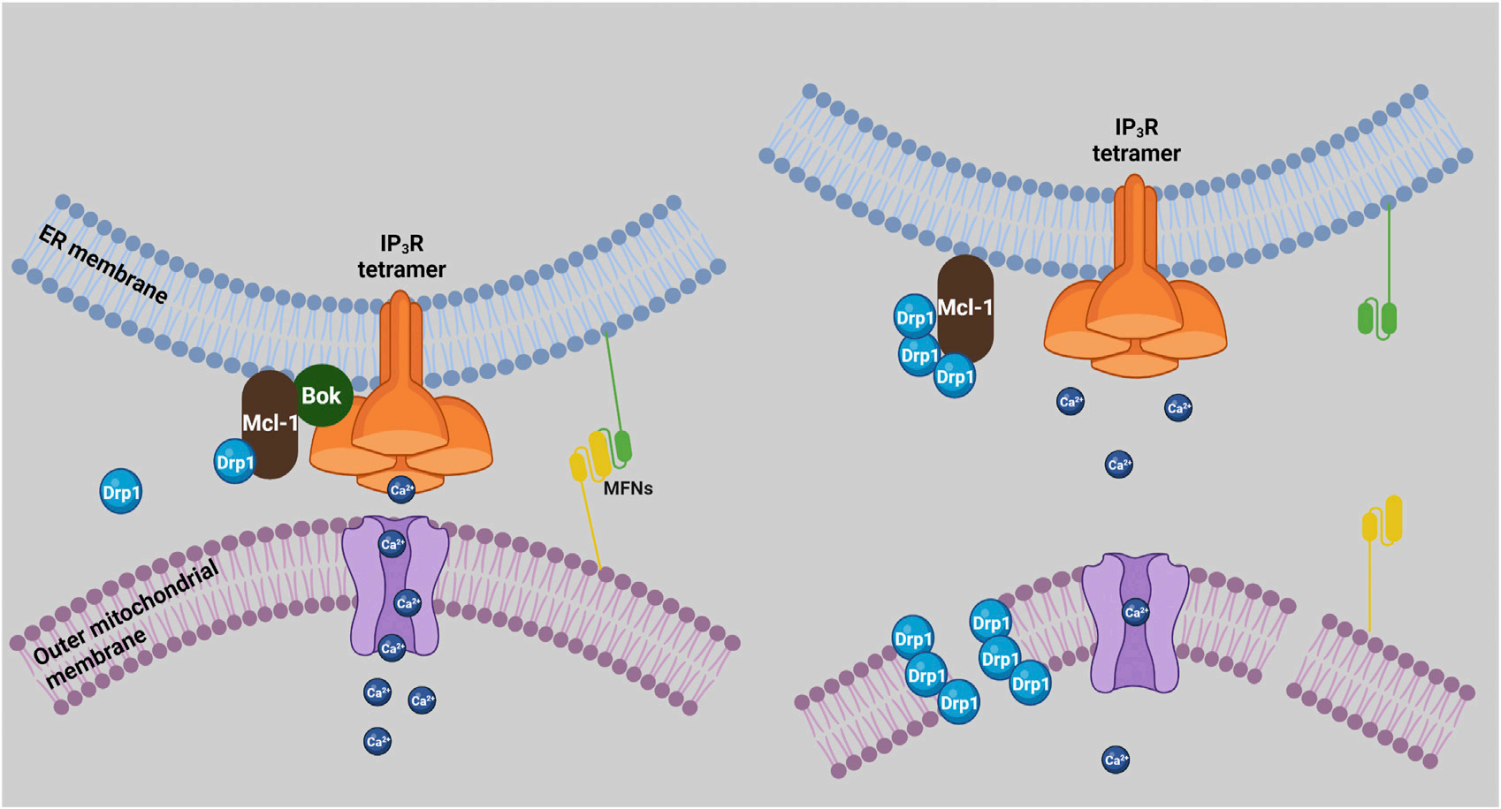
Bok regulation of MAMs and mitochondrial dynamics. In the presence of Bok (left), mitochondria and ER are in close proximity and form MAMs, mitofusins (MFNs) mediate ER/mitochondria tethering, Ca^2+^ is efficiently transferred to mitochondria, and Mcl-1 promotes Drp1 activity, which could be held in check by the ability of Mcl-1 to bind Bok. In the absence of Bok (right), mitochondria and ER separate, mitochondrial uptake of Ca^2+^ is impaired, and mitochondria become fragmented, due to more fission activity and/or less fusion activity. Enhanced fission could result from less Bok antagonism of Mcl-1 leading to more Drp1 activity, while less fusion could result from disruption of mitofusin interactions and activity.

The ER and mitochondria are connected physically at junctions termed MAMs. The many proteins that are present in MAMs mediate a variety of functions, including ER stress responses, Ca^2+^ transfer from ER to mitochondria, and mitochondrial dynamics (Marchi et al., 2014; Carpio et al., 2021; Morris et al., 2021). Recently, it was shown that there is decrease in the proximity and number of ER/mitochondria contact sites in MEFs derived from Bok KO mice (Carpio et al., 2021). This coincided with a decrease in 1) the levels of several MAM proteins, surprisingly including IP_3_R1 and IP_3_R3, 2) Ca^2+^ transfer from ER to mitochondria, and 3) ER stress-induced apoptosis (Carpio et al., 2021). However, reduced IP_3_R levels and Ca^2+^ transfer deficits were not seen in Bok KO MEFs, αT3, and HeLa cells derived by CRISPR-Cas9-mediated gene editing (Schulman et al., 2016; Schulman et al., 2019; Szczesniak et al., 2021b), indicating that these MAM alterations may not be universal. Nevertheless, these findings suggest that a “bystander” role of Bok could be to maintain MAM integrity (Figure 2).

### Bok interaction with non-IP_3_R proteins

Bok was first discovered in a rat ovarian fusion cDNA library using Mcl-1 as bait (Hsu et al., 1997). Mcl-1 is a well-characterized anti-apoptotic protein, with additional non-apoptotic roles, and is upregulated in many cancer cells making it an attractive drug target (Gross and Katz, 2017; Xiang et al., 2018). Recently, studies using TurboID (proximity-dependent biotin identification), revealed Mcl-1 as the only Bcl-2 family member in close proximity to Bok (Szczesniak et al., 2021b). The putative Bok/Mcl-1 interaction appears to be dependent on their TMDs (Stehle et al., 2018; Lucendo et al., 2020; Szczesniak et al., 2021b) and surprisingly, fine-tuned by atypical positively-charged amino acids (Arginine200 and Lysine203) found in the Bok TMD (Bonzerato et al., 2022). Although an interaction between endogenous Bok and Mcl-1 has not yet been clearly demonstrated (Szczesniak et al., 2021b), it is plausible that the ability of exogenous Bok to induce apoptosis is due to antagonism of the anti-apoptotic effects of endogenous Mcl-1, rather than exogenous Bok directly causing MOMP (Stehle et al., 2018; Bonzerato et al., 2022).

Bok has also been shown to interact with and increase the activity of uridine monophosphate synthetase (UMPS), a crucial enzyme for uridine metabolism and nucleotide synthesis. Bok KO cells have reduced UMPS activity, leading to a decrease in cell proliferation (Rabachini et al., 2018) and resistance to 5-fluorouracil (5-FU), since UMPS converts 5-FU to its toxic metabolites (Srivastava et al., 2019; Naim and Kaufmann, 2020). UMPS is predominately a cytosolic enzyme; however, it was also shown to co-localize with Bok suggesting that the Bok/UMPS interaction occurs at the ER membrane (Loffler et al., 2005; Srivastava et al., 2019).

### Bok in mitochondrial dynamics

Maintaining mitochondrial morphology is crucial for cell health and is governed by the balance between fission and fusion (Aouacheria et al., 2017). Bcl-2 family members are key regulators of these processes, with Mcl-1 known to act as a fission regulator, and Bax as a fusion regulator (Hardwick and Soane, 2013; Aouacheria et al., 2017; Rasmussen and Gama, 2020). Mcl-1 interacts with and activates Drp1, promoting mitochondrial fission, while Bax stimulates mitofusin 2 activity, promoting mitochondrial fusion (Hoppins et al., 2011; Rasmussen et al., 2018). Regarding Bok, various Bok KO cell types have fragmented mitochondria, for reasons currently unknown (Schulman et al., 2019; Szczesniak et al., 2021b). Interestingly, TurboID studies show that Bok is in close proximity to many fission mediators (e.g., Drp1, Inf2, etc.), rather than fusion mediators (Szczesniak et al., 2021b). Since Bok appears to bind to and may antagonize Mcl-1 activity, it is plausible that Bok KO could enhance the ability of Mcl-1 to activate Drp1, leading to more mitochondrial fission and fragmentation (Figure 2).

It is also possible that the role of Bok in maintaining MAM integrity (Carpio et al., 2021) impacts mitochondrial dynamics, since it is well-established that mitochondrial fission and fusion are regulated at ER/mitochondria junctions (Abrisch et al., 2020; Means and Katz, 2021). Indeed, tethering of mitochondria to ER is mediated by mitofusins (de Brito and Scorrano, 2008; Naon et al., 2016), and disruption of MAMs in Bok KO cells could reduce mitofusin activity, leading to mitochondrial fragmentation. Whatever the mechanism, it is clear that Bok plays a “bystander” role in maintaining mitochondrial morphology (Figure 2).

## Conclusion

There is no doubt that over-expressed, exogenous Bok can act as a “killer”, but the preponderance of evidence indicates that endogenous Bok, which is only stable when bound to IP_3_Rs, is a “bystander”, with non-apoptotic roles in the cell. In particular, ER/mitochondrial contact sites and mitochondrial dynamics appear to be controlled by endogenous Bok. It is curious that even though exogenous Bok can act similarly to the *bona fide* killers Bak and Bax, endogenous Bok does not act lethally in a native context. Was endogenous Bok originally a MOMP mediator, like Bak and Bax, or was it always an IP_3_R-bound protein with non-apoptotic roles? Was having three active killers too detrimental to cell health and therefore evolution has repurposed Bok to have non-lethal, but still essential roles in the cell? Finally, and most importantly, we hope that these efforts to understand the properties of endogenous and exogenous Bok will provide a better understanding of the Bcl-2 family and potentially identify new therapeutic avenues.

## Abbreviations

Bok, Bcl-2-related ovarian killer; Ca^2+^, calcium, K_d_, dissociation constant; Drp1, dynamin-1-like protein; ER, endoplasmic reticulum; IP_3_R, inositol 1,4,5-trisphosphate receptor; KO, knock-out; MAMs, mitochondria-associated ER membranes; MEF, mouse embryonic fibroblast; MOM, mitochondrial outer membrane; MOMP, mitochondrial outer membrane permeabilization; Mcl-1, myeloid-cell leukemia 1; TA, tail-anchored; TMD, transmembrane domain; E2, ubiquitin-conjugating enzyme; E3, ubiquitin-ligase enzyme; UPP, ubiquitin-proteasome pathway.
